# Data supporting the understanding of modulatory function of opioid analgesics in mouse macrophage activity

**DOI:** 10.1016/j.dib.2017.12.017

**Published:** 2017-12-13

**Authors:** Iwona Filipczak-Bryniarska, Katarzyna Nazimek, Bernadeta Nowak, Michael Kozlowski, Magdalena Wąsik, Krzysztof Bryniarski

**Affiliations:** a Department of Pain Treatment and Palliative Care, Jagiellonian University Medical College, 10 Sniadeckich St, PL 31-531 Krakow, Poland; b Department of Immunology, Jagiellonian University Medical College, 18 Czysta St, PL 31-121 Krakow, Poland

**Keywords:** Immune modulation, Macrophages, Opioids, Phagocytosis

## Abstract

The data presented herein expand the current understanding of the modulatory function of opioid drugs in mouse macrophage activity described in our relevant research article (Filipczak-Bryniarska et al., 2017) [1], in which we characterize the influence of morphine, buprenorphine and oxycodone on humoral and cell-mediated immune response in mice. Among other things, we have shown the effects of treatment with assayed analgesics on macrophage ability to induce antigen-specific B-cell response to sheep red blood cells as well as to generate reactive oxygen intermediates and nitric oxide. The current data demonstrate the effects of morphine, buprenorphine or oxycodone administration on phagocytosis of sheep red blood cells and zymosan by mouse macrophages, supplementing the data on immune modulatory capacities of assayed drugs, recently reported by us (Filipczak-Bryniarska et al., 2017; Kozlowski et al., 2017) [1,2].

**Specifications Table**

**Table:** 

Subject area	Immunology
More specific subject area	Immunopharmacology
Type of data	Figure
How data was acquired	Using flow cytometry on FACS Calibur with BD CellQuest Pro software (BD Bioscience, San Jose, CA, USA)
Data format	Analyzed
Experimental factors	– Treatment of mouse donors of macrophages with proper opioid drug – Injection of mineral oil for induction of macrophage-enriched peritoneal exudate – Collection of macrophages for in vitro incubation with either FITC-coupled sheep red blood cells or zymosan-green – Cytometric analysis of macrophages
Experimental features	– Mouse donors of macrophages were treated for 7 consecutive days with one of tested opioid drugs – Macrophage-enriched peritoneal exudate was induced by mineral oil injection on 2nd day of drug treatment – Yielded macrophages were incubated with either ex tempore FITC-coupled sheep red blood cells or commercially available zymosan-green – Macrophages were analyzed cytometrically for the intensity of green fluorescence emitted
Data source location	Department of Immunology, Jagiellonian University Medical College, Krakow, Poland
Data accessibility	Data is within this article

**Value of the data**

• The data presented here demonstrate the effects of morphine, buprenorphine or oxycodone administration on phagocytosis of either FITC-coupled sheep red blood cells or zymosan-green by mouse macrophages.
• While zymosan-green reagent is commercially available, we have elaborated another, original phagocytosis assay with the use of FITC-coupled sheep red blood cells, prepared by us *ex tempore.*
• The opioid analgesics were administered *in vivo* prior to macrophage harvest, which enables to evaluate their influence on immune cells taking into account the pharmacodynamics, tissue distribution and metabolism of tested drugs in living organism.

## 1 Data

**Fig. 1:**
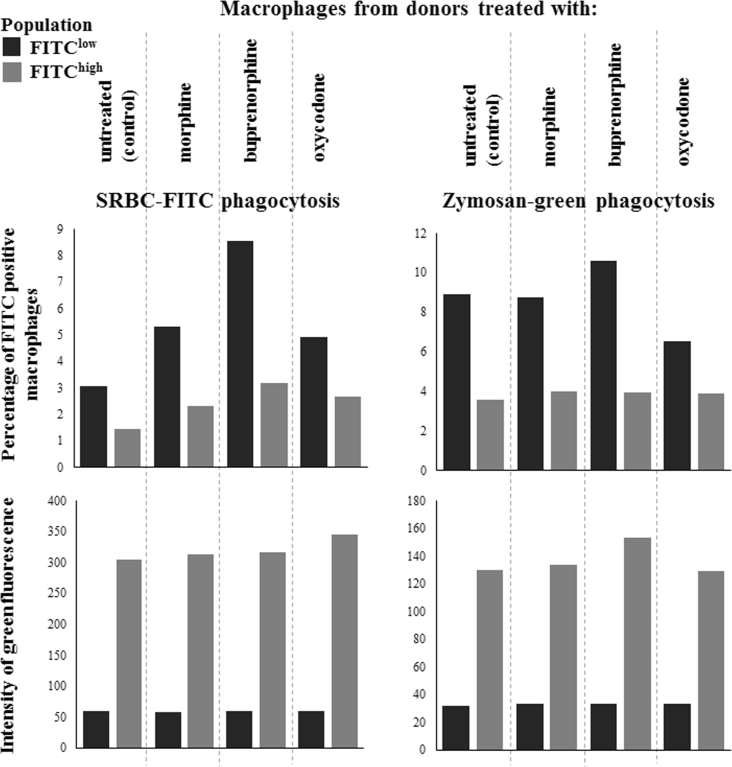
Opioid analgesics influence phagocytosis of fluorescein isothiocyanate (FITC)-coupled sheep red blood cells (SRBC) and zymosan-green by mouse macrophages. Oil-induced, peritoneal macrophages harvested from mice treated for a week with morphine, buprenorphine or oxycodone were incubated for 20 min in 37 °C water-bath with SRBC-FITC or zymosan-green reagent, and then green fluorescence emission by these cells was measured cytometrically. Upper left graph shows the percentage of macrophages of mice treated with respective opioid drug that emit green fluorescence after incubation with SRBC-FITC, while upper right graph shows the percentage of macrophages emitting green fluorescence after incubation with zymosan-green reagent. Lower left graph demonstrates the geometric mean of emitted green fluorescence by control and opioid-treated macrophages incubated with SRBC-FITC and lower right graph depicts the geometric mean of emitted green fluorescence by control and opioid-treated macrophages incubated with zymosan-green reagent. According to the intensity of green fluorescence signal, macrophage populations were divided into cells expressing high (FITC^high^, grey bars) or low (FITC^low^, black bars) fluorescence emission.

The percentage of macrophages from mice treated with different opioid drugs emitting green fluorescence after incubation with fluorescein isothiocyanate (FITC)-coupled sheep red blood cells (Fig. 1, upper left graph) or with zymosan-green reagent (Fig. 1, upper right graph), and the geometric mean of emitted green fluorescence by these macrophages incubated with FITC-coupled sheep red blood cells (Fig. 1, lower left graph) or with zymosan-green reagent (Fig. 1, lower right graph). In addition, macrophage populations were divided into cells expressing high (FITC^high^, grey bars) or low (FITC^low^, black bars) green fluorescence emission. The raw data are included in supplementary table.

## 2 Experimental design, materials and methods

### 2.1 Mice

Ten week old male mice (22 ± 2 g) of the inbred CBA strain were from the breeding unit of the Department of Immunology, Faculty of Medicine, Jagiellonian University Medical College, Krakow, Poland and were fed autoclaved food and water *ad libitum*. After random assignment to control or treatment groups, animals were subjected to the experiment conducted according to the guidelines of the 1st Local Ethics Committee in Krakow (approval No 123/2013).

### 2.2 Opioid drug administration, mineral oil injection and harvest of macrophages

Mice were treated intraperitoneally for 7 consecutive days with sterile Dulbecco's phosphate buffered saline (DPBS) solutions of following drugs: morphine sulfate (02DR0910, WZF Polfa S.A., Warsaw, Poland) administered twice daily in a single dose of 20 mg/kg per mouse [1], [2], [3], buprenorphine (01AF0512, WZF Polfa S.A., Warsaw, Poland) given once a day in a dose of 2 mg/kg per mouse [1], [2], and oxycodone hydrochloride (AB465, Mundipharma Polska Sp. z o.o., Warsaw, Poland) administered twice daily in a single dose of 20 mg/kg per mouse [1], [2]. To induce macrophage-enriched peritoneal exudate, on the 2nd day of drug administration mice were injected intraperitoneally with 1 ml of mineral oil heavy fraction (Sigma, St. Louis, MO, USA) [1], [2], [3]. Five days later peritoneal exudate containing over 95% of macrophages was harvested by washing the peritoneal cavity with ice-cold DPBS containing heparin (5 U/ml) [1], [2], [3] and, after washing, macrophages were used in the *in vitro* phagocytosis assay.

### 2.3 Preparation of FITC-coupled sheep red blood cells (SRBC)

Powdered FITC (Sigma, St. Louis, MO, USA) was dissolved in Tris-HCl buffer (pH 9.0) to reach the concentration of 50 mg/ml. Then it was diluted 10 times with DPBS, adjusted to pH 8.0, and 10 ml of the final FITC solution was mixed with 1 ml of 100% suspension of SRBC (Graso Biotech, Starogard Gdanski, Poland), washed previously with DPBS, and incubated for 2 h at room temperature, in darkness, on hematological roller. Afterwards, SRBC-FITC were washed thrice with DPBS, filtered through nylon mesh and 1% suspension in DPBS was prepared for the phagocytosis assay.

### 2.4 Phagocytosis assay and cytometric analysis

Macrophages harvested from either drug-treated or untreated (control) mice were incubated with SRBC-FITC (in ratio 1:10) for 20 min in 37 °C water-bath, which was followed with osmotic shock for lysis and removal of non-phagocytosed SRBC-FITC. Otherwise, macrophages were incubated with zymosan-green reagent (pHrodo^TM^ Green Zymosan A BioParticles Conjugate; Life Technologies, Carlsbad, CA, USA), previously prepared according to manufacturer procedures, in a dose of 0.25 mg of zymosan per 5 × 10^6^ macrophages for 20 min in 37 °C water-bath. Then cells were analyzed by flow cytometry on FACS Calibur (BD Bioscience, San Jose, CA, USA), and acquired data were analyzed firstly with BD CellQuest Pro software, and then with GraphPad Prism and Excel software.
