# Supplementary material for: Data supporting the understanding of modulatory function of opioid analgesics in mouse macrophage activity
Source: Data Brief. 2017 Dec 13;16:950–4. doi: 10.1016/j.dib.2017.12.017 (PMC5751875; doi:10.1016/j.dib.2017.12.017)
Supplement: Supplementary file 1 — Transparency document [file mmc1.pdf]

JAGIELLONIAN UNIVERSITY MEDICAL COLLEGE
Department of Immunology
31-121 Kraków, ul. Czysta 18
tel./fax: +48 (12) 633-94-31

December 1, 2017
Krakow, Poland

Reference: Manuscript " **Data supporting the understanding of modulatory function of opioid analgesics in mouse macrophage activity"** by Iwona Filipczak-Bryniarska, Katarzyna Nazimek, Bernadeta Nowak, Michael Kozlowski, Magdalena Wąsik and Krzysztof Bryniarski, submitted for consideration as a *data paper* in ***Data in Brief (DiB)*** journal.

There are no conflicts of interest associated with this work.

All correspondence should be sent to Krzysztof Bryniarski,
krzysztof.bryniarski@uj.edu.pl
