# Supplementary material for: Data supporting the understanding of modulatory function of opioid analgesics in mouse macrophage activity
Source: Data Brief. 2017 Dec 13;16:950–4. doi: 10.1016/j.dib.2017.12.017 (PMC5751875; doi:10.1016/j.dib.2017.12.017)
Supplement: Supplementary file 2 — Supplementary material [file mmc2.pdf]

Supplementary table: raw data from the study on opioid analgesics influence on phagocytosis of fluorescein isothiocyanate (FITC)-coupled sheep red blood cells (SRBC) and zymosan-green by mouse macrophages.

Donors of macrophages were treated with:	Phagocytosis of SRBC-FITC				Phagocytosis of zymosan-green			
	% of FITC fluorescence emitting cells		Intensity of FITC fluorescence emission		% of FITC fluorescence emitting cells		Intensity of FITC fluorescence emission	
	$FITC^{low}$	$FITC^{high}$	$FITC^{low}$	$FITC^{high}$	$FITC^{low}$	$FITC^{high}$	$FITC^{low}$	$FITC^{high}$
untreated (control)	3,05	1,45	59	304	8,93	3,56	32	130
morphine	5,29	2,31	58	314	8,77	4,00	33	134
buprenorphine	8,56	3,19	59	316	10,61	3,97	33	153
oxycodone	4,92	2,68	59	345	6,54	3,91	33	129
	percentage		geometric mean		percentage		geometric mean	
